# *Cytidine monophospho-N-acetylneuraminic acid hydroxylase *(*CMAH*) mutations associated with the domestic cat AB blood group

**DOI:** 10.1186/1471-2156-8-27

**Published:** 2007-06-06

**Authors:** Barbara Bighignoli, Tirri Niini, Robert A Grahn, Niels C Pedersen, Lee V Millon, Michele Polli, Maria Longeri, Leslie A Lyons

**Affiliations:** 1 Istituto di Zootecnica, Faculty of Veterinary Medicine, University of Milan, Milan, Italy; 2 Oy Triniini Company, P.O. Box 36, FIN-00501, Helsinki, Finland; 3 Department of Population Health and Reproduction, School of Veterinary Medicine, University of California, Davis, Davis, CA USA; 4 Department of Veterinary Medicine and Epidemiology, School of Veterinary Medicine, University of California, Davis, Davis, CA USA; 5 Veterinary Genetics Laboratory, School of Veterinary Medicine, University of California, Davis, Davis, CA USA

## Abstract

**Background:**

The cat has one common blood group with two major serotypes, blood type A that is dominant to type B. A rare type AB may also be allelic and is suspected to be recessive to A and dominant to B. Cat blood type antigens are defined, N-glycolylneuraminic acid (NeuGc) is associated with type A and N-acetylneuraminic acid (NeuAc) with type B. The enzyme *cytidine monophospho-N-acetylneuraminic acid hydroxylase *(*CMAH*) determines the sugar bound to the red cell by converting NeuAc to NeuGc. Thus, mutations in *CMAH *may cause the A and B blood types.

**Results:**

Genomic sequence of *CMAH *from eight cats and the cDNA of four cats representing all blood types were analyzed to identify causative mutations. DNA variants consistent with the blood types were genotyped in over 200 cats. Five SNPs and an indel formed haplotypes that were consistent with each blood type.

**Conclusion:**

Mutations in type B cats likely disrupt the gene function of *CMAH*, leading to a predominance of NeuAc. Type AB concordant variants were not identified, however, cDNA species suggest an alternative allele that activates a downstream start site, leading to a CMAH protein that would be altered at the 5' region. The cat AB blood group system is proposed to be designated by three alleles, *A *> *a*^*ab *^> *b*. The *A *and *b CMAH *alleles described herein can distinguish type A and type B cats without blood sample collections. *CMAH *represents the first blood group gene identified outside of non-human primates and humans.

## Background

Only one major blood group system has been identified for the domestic cat, whereas a plethora of systems are recognized in dogs, humans, horses and other species [1]. Except in primates, no gene mutations have been identified that cause the many animal blood groups. The major blood group system of the domestic cat was identified in the early 1900's [2,3] and was later found to contain two types as determined by agglutination by naturally occurring alloantibodies [4,5]. The two major blood types are called type A and type B [6]. The common types are allelic, with type A dominant to type B. A rare type AB, which shows agglutination with both anti-A and anti-B, has not been as clearly defined, but is suspected to be allelic to types A and B [6,7]. Several studies have shown familial segregation for cat blood types A and B. However, families segregating for type AB are limited and the parentage of the matings was not confirmed. Therefore, the inheritance of the third type AB allele was not clearly resolved [6,7], although it appears to be recessive to type A and dominant to type B [8].

A majority of random bred cats throughout the world are type A [4-6,9-13]. However, some cat breeds have a high frequency of type B individuals. The Siamese, Tonkinese and Burmese breeds are nearly fixed for type A, whereas the prevalence of type B individuals of British Shorthairs, Birmans, Devon and Cornish Rex can range between 25 – 50% [14]. Although type B cats are rare in random bred populations, admixtures with cat breeds are increasing the frequency of type B cats in the random bred population. For example, type B cats are more common in the random bred populations of California [15] and Australia than in Europe and the rest of the United States [13]. Thus, veterinary clinics and hospitals are finding it increasingly necessary to maintain both type A and type B cats for blood transfusions.

The ability to determine feline blood types is especially important because of the phenomena of neonatal isoerythrolysis [16-18] and transfusion reactions [19-22] between previously non-sensitized donors. Similar to the unrelated ABO system of humans, cats often possess alloantibodies against their opposite blood type. Blood type B cats possess strong agglutinins and hemolysins to type A red cells. Type A cats possess alloantibodies that are less strongly reactive for type B red cells. Thus, a blood transfusion of a type B cat with type A blood can cause a severe reaction. Milder reactions occur if type B blood is transfused into a type A cat. Interestingly, blood type AB cats are universal recipients since they lack alloantibodies against either blood type. The presence of strong, naturally occurring alloantibodies in the type B cat against the A antigens also leads to neonatal isoerythrolysis for type A kittens born from a type B queen. The prevention of transfusion reactions and neonatal isoerythrolysis requires close monitoring of blood types of donor and recipient, and toms and queens [17,23-26].

The cause and the likely enzymatic mechanism for the cat blood groups is known. The A and B blood types of cats are caused by differences in the neuraminic acid residues present on a ceramide dihexose backbone on the surface of erythrocytes [27-30]. The type A cat has mainly N-glycolylneuraminic acid (NeuGc) and a small amount of N-acetylneuraminic acid (NeuAc), while the type B cat has only NeuAc [28]. Since A positive is dominant to B positive, obligate carriers of the B blood type present similarly to the common A positive cats. Rare type AB positive cats have both NeuAc and NeuGc presented on the red cells in similar quantities, each at approximately 50% normal expression levels [7].

NeuAc is converted to NeuGc in a pathway that is catalyzed by *cytidine monophospho-N-acetylneuraminic acid hydroxylase *(*CMAH*) [31]. N-glycolylneuraminic acid (NeuGc) is expressed in most mammals, but not in humans [32]. Human *CMAH *was inactivated after their divergence from chimpanzees by a deletion in the coding region [32]. *CMAH *is postulated to be active only in type A red cells and either absent or nonfunctional in type B red cells, because recessive type B cats do not convert NeuAc to NeuGc [28]. In this study, feline *CMAH *was characterized and examined for mutations that could influence and characterize the cat AB blood group system.

## Results

### Genomic and mRNA analyses

The 2× trace sequence of the cat [UCSC Genome Browser scaffold_185890, 185896, 215984] [33] and the *CMAH *sequence from the domestic dog were sufficient to identify twelve of the putative 14 exons of feline *CMAH*. Cat *CMAH *exons 4 and 10 were absent from the trace archives, however, coding region and UTR sequences were subsequently obtained from the cDNA studies. The complete *CMAH *cDNA was sequenced from four cats, including a type A Oriental Shorthair [GenBank: EF127684], a type B Persian [GenBank: EF127685], a type AB Siamese and a crossbred heterozygous B carrier (Type Ab). The cDNA sequence and protein translation are presented in Additional file 1. The cDNA sequences were compared with published sequences from several species, including mouse [GenBank: D21826], human [GenBank: AF074480], dog [Ensembl: ENSCAFT00000016708] and cat. Assuming the putative start methionine is the next to last codon of exon 1, the cat *CMAH *had 578 amino acids and 14 coded exons, producing a 1734 nucleotide transcript [Additional file 1]. Only the last 6 bps of exon 1 were translated, presenting the putative start codon, methionine, and a glycine, prior to the splice to the fully translated exon 2, which began with a serine. The first 72 amino acids in terminal exon 14 were translated; the polyadenylation signal is 373 bp downstream of the stop codon. Cat *CMAH *exhibited 91.7% nucleotide sequence identity to the predicted sequence of the dog, 83.7% with the mouse, and 84.5% with human. At the protein level, cat *CMAH *was 92.9% identical to the dog, 89.6% identical to the mouse and 87.7% identical to human.

**Figure 1:**
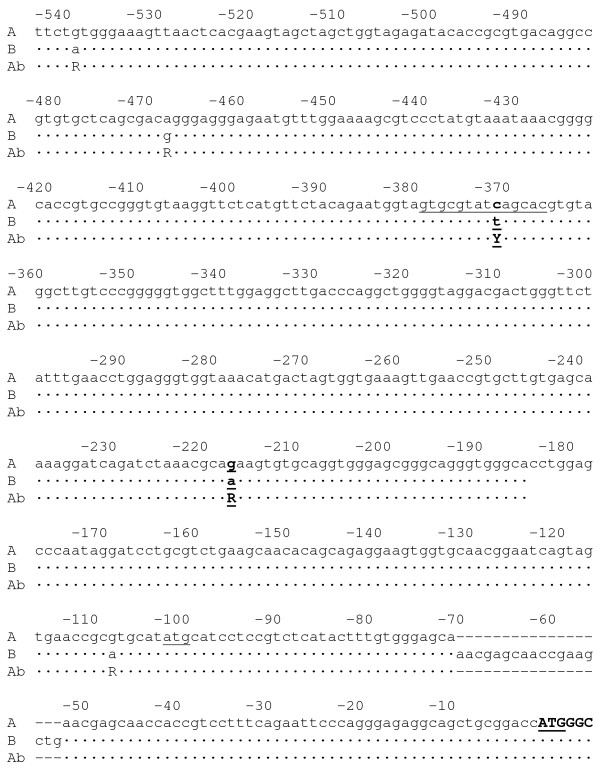
**Cat genomic sequence of *CMAH *including the 5'UTR to exon 1**. Sequence generated from genomic DNA from cats of each blood type. Lower case letters represent the UTR. The six bold upper case letters are the last six nucleotides of exon 1 that are translated to the methionine start and a glycine prior to the splice with exon 2. ATG sites are underlined. The lower case in italics is the intronic 1 sequence that is the splice variant sequence found in the cDNA of Type B cats, spliced immediately upstream of the exon 2 sequence. Two GATA transcription factor sites are underlined. The mutations that are consistent with blood group phenotypes, A-217G and the T-371C, are presented in bold and underlined. One intron 1 variant and three 5' UTR mutations that are not consistent with blood types are also presented.

Sequence analyses of the coding regions identified eleven DNA variants including seven silent and four missense mutations [Additional file 1]. The missense mutations included an exon 2 G139A (V47M), an exon 2 T265A (Y89N), an exon 4 A324C (E108D) and an exon 13 G1600A (D534N). Approximately 550 bp of genomic DNA sequence were analyzed upstream of the start codon in exon 1 (Figure 1), including the exon 1 5' UTR, in six type A cats, three type B cats and two type AB cats [Type A UTR GenBank: EF127683], [Type B GenBank: EF127686]. Five additional SNPs were identified, including G-108A, G-217A, C-371T, A-468G, and G-539A, and an 18 bp indel at position -53 in the 5' UTR (Figure 1).

**Figure 2:**
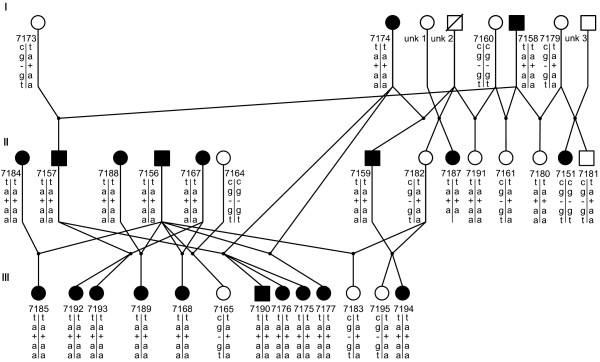
**Pedigree of a British Shorthair cat family segregating for blood types and *CMAH *variants**. Circles represent females, squares represent males, filled symbols indicate type B cats from hemagglutination assays. Symbols with a diagonal slash represent cats that were not available for the analyses. Numbers under the symbols represent the laboratory sample number. Genotypes for the *CMAH *variants are represented below the cat identification numbers, respectively, and are presented as haplotypes in the order; G-217A, T-371C, Δ-53, G139A, T265A.

**Figure 3:**
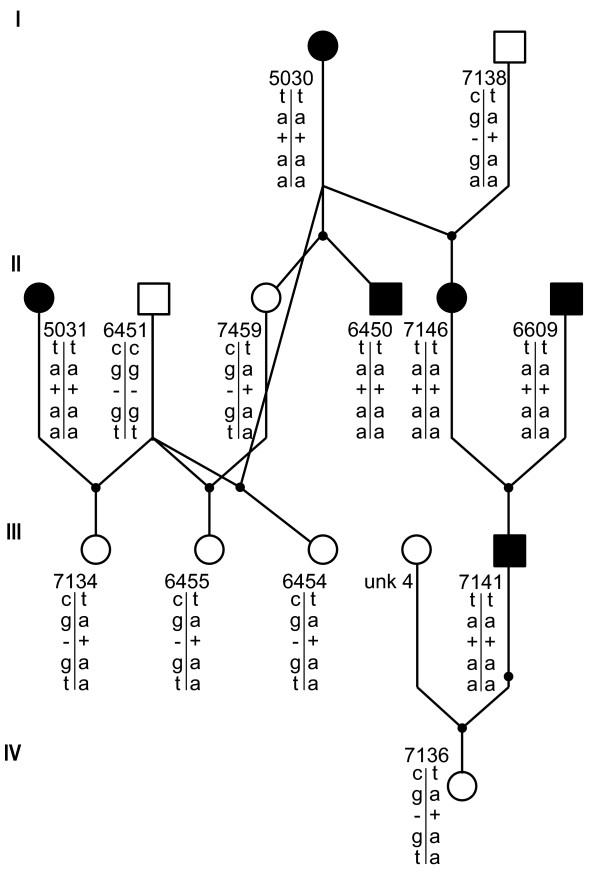
**Pedigree of a Birman cat family segregating for blood types and *CMAH *variants**. Symbols, numbering and haplotype order are the same as described for Figure 2.

Two upstream SNPs, G-217A and C-371T, the 18 bp indel, three missense coding region SNPs, G139A, T265A, and G1600A and the silent G1266A in exon 11 were consistent with the blood types in the eight sequenced cats and were subsequently genotyped in 213 additional cats (Table 1), which included the cats comprising the two purebred pedigrees (Figures 2, 3). The 18 bp indel was deleted in the type A homozygous cats, but inserted in the type B cat. All type B cats were homozygous for the identified variants. All type A cats that were obligate carriers of type B were heterozygous, suggesting 2 haplotypes controlling the cat blood types. No cats that were type A were homozygous for the type B haplotype. In addition, these haplotypes segregated concordantly with the blood types in the two breed pedigrees (Figures 2, 3). Only one coding region SNP, the silent G1266A mutation in exon 11, was inconsistent with the blood types in the expanded sample pool. Approximately 20 individuals had genotypes that were discordant with the blood types (data not shown). Flanking sequences were insufficient to genotype the A324C SNP in additional cats. The remaining six silent coding region mutations were not present in all the cats with the same blood type or were unique to an individual cat [Additional file 1]. GenBank accessions represent the sequences of the identified DNA variants consistent with type B cats [GenBank: EF127686].

**Table 1:** *CMAH *DNA variants genotyped in domestic cats.

	**Blood**		**5' UTR**			**E2**	**E3**	**E13**
						
**Breed**	**Type**^1^	**No**.	**A-217G**	**C-371T**	**Δ-53**	**G139A**	**T265A**	**G1600A**
Trace archive			A	A	N	G	T	G

Birman^3^	A	18	AA	CC	NN	GG	TT	GG
British SH^3^	A	5	AA	CC	NN	GG	TT	GG
Devon Rex^3^	A	5	AA	CC	NN	GG	TT	GG
Domestic SH^3^	A	1	AA	CC	NN	GG	TT	GG
Exotic SH	A	3	AA	CC	NN	GG	TT	GG
Oriental SH^4^	A	1	AA	CC	NN	GG	TT	GG
Scottish Fold	A	2	AA	CC	NN	GG	TT	GG

Type A Haplotype			A	C	N	G	T	G

Birman	A	19	AG	CT	NP	GA	TA	GA
British SH	A	14	AG	CT	NP	GA	TA	GA
Devon Rex	A	18	AG	CT	NP	GA	TA	GA
Domestic SH	A	1	AG	CT	NP	GA	TA	GA
Exotic SH	A	1	AG	CT	NP	GA	TA	GA
Hybrid^4^	Ab	12	AG	CT	NP	GA	TA	GA
Scottish Fold	A	5	AG	CT	NP	GA	TA	GA
Siberian	A	2	AG	CT	NP	GA	TA	GA
Birman^3^	B	22	GG	TT	PP	AA	AA	AA
British SH^3^	B	30	GG	TT	PP	AA	AA	AA
Devon Rex	B	25	GG	TT	PP	AA	AA	AA
Persian^4^	B	1	GG	TT	PP	AA	AA	AA
Scottish Fold	B	1	GG	TT	PP	AA	AA	AA

Type B haplotype			G	T	P	A	A	A

Bengal	AB	1	AA	CC	NN	GG	TT	GG
Devon Rex	AB	1	AA	CC	NN	GG	TT	GG
Egyptian Mau	AB	1	AA	CC	NN	GG	TT	GG
European	AB	4	AA	CC	NN	GG	TT	GG
Ragdoll	AB	8	AA	CC	NN	GG	TT	GG
Siamese^4^	AB	1	AA	CC	NN	GG	TT	GG
Siberian	AB	1	AA	CC	NN	GG	TT	GG
British SH	AB	3	AG	CT	NP	GA	TA	GA
Cornish Rex	AB	2	AG	CT	NP	GA	TA	GA
Devon Rex	AB	7	AG	CT	NP	GA	TA	GA
European	AB	1	AG	CT	NP	GA	TA	GA
Maine Coon	AB	1	AG	CT	NP	GA	TA	GA
Manx	AB	1	AG	CT	NP	GA	TA	GA
Ragdoll^3^	AB	1	AG	CT	NP	GA	TA	GA
Sphynx^3^	AB	2	AG	CT	NN	GA	TA	GA

**Totals**: 18 breeds		N = 221						

The column labels indicate which region of the gene had the mutation, such as the 5' UTR or exons 2, 3, or 13. The actual base pair changes and positions are noted above each column. The delta symbol indicates a deletion. The sequence for the trace archive sequence from GenBank of the Abyssinian cat is listed first. 1. Blood typing was determined as described in the Methods. 2. The 18 bp indel is represented by N for not present and P for present. All other mutations are presented as their nucleotide abbreviation (A = adenine, C = cytosine, T = thymidine, G = guanine). 3. The genomic sequence of each *CMAH *exon, except exons 4 and 10 was determined for one cat of each of the indicated breeds. 4. The cDNA sequence was determined for one cat of each of the indicated breeds.

### 5' UTR mRNA analysis

**Figure 4:**
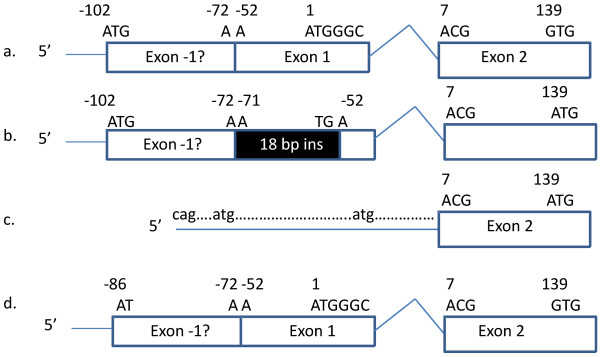
**Schematic of putative cat CMAH protein deduced from mRNA**. a. The ATG at -102 bp may be the true activation site in the cat, implying an extension to exon 1 exists in the cat, 5' to *CMAH *exon 1 that has been declared in mouse *Cmah*. Blood type A cats have at least one allele that creates the depicted fully functional protein. b. An 18 bp insertion is homozygous in the genomic sequence of type B cats. This insertion disrupts the reading frame created by the -102 bp ATG, causing a TGA stop codon. Approximately 50% of the mRNA of the type B cat had this insertion. c. Approximately 50% of the mRNA from the blood type B cats had the 3' portion of intron 1 spliced directly to exon 2, completely skipping exon 1, including the insertion. Two ATG sequences are present in this intronic leader but are not in frame. The first in-frame ATG occurs at bp 139 in exon 2 for cats with this mis-spliced mRNA species. The insertion and G139A mutation is concordant with the *b *allele. Cats that are blood type A but carry the *b *allele had all three species of mRNA. d. The mRNA from cats of the rare blood type AB did not extend to the -102 bp start and did not have the 18 bp insertion. The traditional ATG start at bp 1 would be the first in-frame start site. Approximately 50% of the mRNA from the blood type AB cat also had mRNA species as depicted in figure 5c but not the G139A mutation as found on the *b *allele.

The cloned 5' UTR sequences detected more than two species of cDNA sequences in three of the four cats analyzed by mRNA. All of the clones from the homozygous A cat and 50% of the clones from the heterozygous A cat had sequences that extended to approximately -121 bp, which included an in-frame ATG at -102 bp (Figure 4). Approximately 50% of the clones from the type B cat also extended to -121 bp, which included the ATG at -99 bp and the 18 bp insertion at -53 bp. Unexpectedly, the remaining type B cDNA clones were completely missing exon 1. In these particular clones, intron 1 sequence appeared prior to exon 2 sequence, with novel ATGs 16 bp and 59 bp upstream of the splice site for the initial serine codon for exon 2 (Figures 1 &4). Either of these intronic ATGs caused a shift in the reading frame of the protein. The two cDNA forms found in the Type B cat were also identified in the heterozygous A and AB cats, but at a lower frequency.

### Blood type AB cats

Approximately 8,650 samples of cat blood have been typed by hemagglutination assay at the collaborating service laboratory (data not shown). Approximately 133 cats were type AB, suggesting a frequency of approximately 1.5% in the European cat population. However, a majority of the type AB cats represented the Ragdoll breed (N = 104) and European shorthairs (N = 18). Approximately 50% of cDNA sequences from the type AB cat were missing exon 1 and had intron 1 sequence upstream of exon 2, similar to the type B cats. Blood type AB cats were never homozygous for the haplotype of the B blood group; they were either homozygous or heterozygous *A*. Unexpectedly, two type AB cats of the Sphynx breed were homozygous for the 18 bp insertion, but heterozygous for the four other mutations in the haplotypes (Table 1).

## Discussion

Dozens of blood group systems have been recognized by the International Society of Blood Transfusion and 39 are genetically characterized in humans [34,35]. Extensive polymorphism is present in some blood groups of humans and some agricultural species, allowing blood groups for use in identification and parentage testing. In addition to pedigree testing, blood typing has been of crucial importance in preventing transfusion reactions and neonatal isoerythrolysis in a variety of species.

The cat AB blood group system is both unique and typical of blood groups of other species. It is unique because it appears to be the sole major blood group system. Since it has only two common alleles and the vast majority of cats have the same blood type A, the cat blood group system is also somewhat unique in not being useful for parentage and identification testing. It is typical of other animal blood groups in that cats with one blood type naturally produce alloantibodies to the opposing blood type [17,26]. Therefore, like other blood group systems, such as ABO of humans, the AB system of cats is important in transfusion reactions and neonatal isoerythrolysis [17,26].

The cat AB blood group is coded by a unique gene, which is not listed among the 39 blood type genes that have been identified in humans. *Cytidine monophospho-N-acetylneuraminic acid hydroxylase (CMAH)*, which is associated with the production of the sialic acids on cat red cells [28], is non-functional in humans and chickens [32,36]. However, *CMAH *has been highly conserved and is active in species as primitive as echinoderms [37]. The present study demonstrated that the genomic structure of cat *CMAH *was similar to the homologous gene in other species. At first, *in silico *comparison to mouse and rat *CMAH *suggested that in the cat, only the last 6 nucleotides of exon 1 and the first 72 in terminal exon 14 were translated.

This study suggests that the cat AB blood group system resulted from mutation(s) in *CMAH *that prevented the conversion of NeuAc to NeuGc, hence type B to type A. Several mutations, including 2 SNPs upstream of the start, an indel in the exon 1 5' UTR and three missense mutations in the coding region were concordant with the type A and type B blood types of cats representing 18 breeds from the USA and Europe. The six mutations were homozygous in all type B cats and heterozygous in all type A cats that were obligate carriers for type B. No type A cats were homozygous for these six variants.

The function of the identified mutations needs to be confirmed by protein studies, however, the functions appear simple in the type A and type B cats (see Figure 4). We hypothesize that *CMAH *may be extended at the amino terminus of the protein as compared to rodents, which would be unique to the cat. For both type A and type B cats, cDNA sequence extended approximately -121 bp upstream of the exon 1 start site. Within this sequence, a potential start codon is located -102 bp upstream of the traditional start site suggested in rodents. Comparative analyses of the -121 bp upstream sequence did not indicate conservation in any other species. For the type A cat, the upstream start would add an additional 28 amino acids to the 5' portion of the gene. In the type B cats, which have an 18 bp indel, the 18 bp insertion generates a stop codon at position -54, which would terminate the protein prior to the translation of exon 1, disrupting nearly the entire protein. Thus, type B cats would not have a functional protein and heterozygous A cats would still have one functional allele, thus allowing full conversion of NeuGC to NeuAC on the red cells. The strength of hemagglutination reactions should be evaluated for correlation with the heterozygous and homozygous A genotypes.

Approximately half of the mRNA sequences from the type B and type AB cats had intron 1 spliced to exon 2. Variants in the 5' upstream region may interact with transcription initiation in the mRNA, leading to the intron 1 sequence that is upstream to exon 2 for approximately 50% of the cDNA species in the type B. Notably, the C-369T mutation disrupts a GATA transcription factor site in the type B cat, which is recognized for erythropoeitic transcription in other species. An additional GATA site is located in intron 1 and is present in both the type A and type B cats [38]. Two ATG sequences are present in this second cDNA variant with intron 1 sequence, however, in frame stop sites occur within a few codons of both sites. The first in frame ATG on the *b *allele would occur at the G139A SNP, which would truncate the amino terminus of the protein by 47 amino acids from the traditional start and by an additional 28 amino acids if the actual start site in the cat is the newly identified ATG at -102 bp. A similar situation would occur in type AB cats with the *Ab *haplotype. The first ATG in frame in a blood type AB cat with an AA haplotype is at M87. Each protein should be highly compromised and likely non-functional.

One of the most intriguing findings of this study concerned the cats that tested positive for both A and B forms of sialic acids. Within the analyzed regions, the blood type AB positive cat could not be differentiated by genomic sequence from a blood type A positive cat. However, no type AB positive cats (N = 26) were homozygous for the type B haplotype. Approximately 50% of the type AB cats have the homozygous *A *haplotype and 50% have the heterozygous *A *haplotype. The blood type AB cat in the mRNA analysis had the homozygous *A *haplotype. Some clones had the type A cDNA form, while some had the intron 1 spliced to exon 2, as found in type B cats. However, the coding region SNPs were homozygous for the *A *haplotype. Since both sialic acids are present on the red cell antigens in the type AB cats [7], *CMAH *must convert some NeuAc to NeuGc, which is different from a blood type A cat carrying a B allele. Although previous studies have not been definitive on the inheritance of Type AB, the present studies indicate that AB is allelic to A and B in cats, and that the unidentified mutation responsible for the AB positive blood type resides on a type *A *allelic background, altering splicing similarly to the type B cats. Thus, we propose a renaming of the cat AB blood group system to contain three alleles represented as *A *> *a*^*ab *^> *b*. The allele *A *would encode the wild-type and fully active enzyme, *a*^*ab *^the enzyme that is partially active, and *b *the inactive enzyme. Possible genotypes/phenotypes would be *AA *(Type A); *Aa*^*ab *^(Type A); *Ab *(Type A); *a*^*ab*^*b *(Type AB), and *a*^*ab*^*a*^*ab *^(Type AB), and *bb *(Type B).

Griot-Wenk *et al*. (1996) have presented strong evidence that type AB of cats is allelic, but the possibility of cis-acting alleles due to unequal recombination or linked loci was not disregarded [6]. Auer and Bell (1981) also hypothesized that the cat AB blood group may be similar to the epistatic effects of the human *H *gene on the human *ABO *system. The lack of variants in *CMAH *that are concordant with the AB blood type also supports the epistatic gene, secondary locus hypothesis. Although a candidate gene cannot be implicated from this study, mutations in other genes of the sialic acid pathway, including *CMP-sialic acid synthetase *(*CMAS*) or perhaps the glycophorins should be considered.

Anomalous findings for the feline AB positive blood type may be explained by a close association between *CMAH *and other blood group genes. Two blood group systems in humans, *I *antigen and *ChidoRogers *(*ChRg*), map to the same genetic region on human chromosome 6p as the defective human *CMAH*. The ChRg blood group system involves the fourth component of complement, while *GCNT2 *encodes beta-1,6-N-acetyl glucosamine transferase, which is responsible for converting i-active straight chains of carbohydrates to I-active branched chains in the *I *antigen system. Cat chromosome B2 corresponded to human chromosome 6 [39], and rearrangements in the short arm of cat chromosome B2, as compared to human chromosome 6p, may have *CMAH *and *GGNT2 *within a linkage group in the cat [39,40]. Therefore, it is possible that *GGNT2 *affects the feline AB positive blood type and explains cosegregation of type AB and B cats in some families and other familial discrepancies. Along with expression studies, more extensive analyses of the *a*^*ab *^allele segregating in families and the analysis of the above implicated genes could resolve the mysterious type AB cat. In addition, two type AB positive cats of the Sphynx breed were found to have the genotype of *b *carriers at all SNPs but not at the indel, where these cats lacked the excepted 18 bp insertion. Thus, these cats warrant further investigation.

## Conclusion

The identified mutations in feline *CMAH *are strongly correlated with cat blood types and may represent the first causative mutations for a blood group in a non-primate species. Genomic and cDNA sequence analyses suggest that the cat exon 1 translation initiates upstream from the putative mouse start codon, generating a protein with 28 additional amino acids at the amino terminus in the cat. Disruption of the reading frame by an 18 bp insertion in exon 1 or an altered splice likely causes enzymatic failure and the production of red cells with NeuGC, which would be the type b allele. Altered splicing and transcription sites may influence the rare, type AB blood group, however, specific mutations were not identified. The present work identifying the causative gene and concordant haplotypes for type A and type B alleles provides a powerful tool to identify *bb *and *b *allele carriers. Until the protein alterations are deduced, genotyping of all or several mutations in the type B haplotype may be warranted to clearly identify *b *carriers. Genetic testing can now help prevent neonatal isoerythrolysis in cat breeding by detecting b carrier cats and preventing the breeding of type B queens bred to type A toms that carry *b*.

## Materials and methods

### Sampling

To maintain clarity within the discussions, cats that are obligate carriers of type B are presented as type Ab, whereas the third, less defined blood group, will be presented in the traditional manner as type AB. Whole blood anti-coagulated with EDTA or buccal swab samples from non-related, as well as related cats with A, B and AB blood types were obtained from owners in the USA. European cat DNA samples, which were received as part of a blood typing service, were also provided by the collaborating service laboratory (Oy Triniini Company, Helsinki, Finland). The complete sample set (N = 221) included 107 type A, 79 type B and 35 type AB cats representing 18 breeds (Table 1). The sample set included; i) 153 purebred cats representing 18 breeds, ii) two purebred pedigrees segregating for the A and B blood types developed for the British Shorthair (N = 31) and Birman (N = 13) cat breeds (Figures 2, 3), iii) twelve obligate carrier F1 hybrid cats produced by crossing a type B Persian with two type A Oriental Shorthairs, thus producing type Ab, and iv) two random bred domestic shorthairs (Table 1). Relationships within the pedigrees were confirmed with a publicly available microsatellite-based parentage testing panel for the cat [41].

Blood types were determined using RapidVet-H Feline Blood Type Cards (DMS Laboratories, Flemington, NJ). Card tests were performed by the investigators or reported by the cat owners. Samples provided by the Oy Triniini Company, which included all except one type AB cat, were typed by hemagglutination and alloantibody assays[6] Briefly, RBCs were isolated from each cat and were independently incubated with anti-A and anti-B sera. Blood types were confirmed by backtyping with the given cat's own plasma to confirm the presence of alloantibodies. DNA from buccal swab samples was isolated using the QIAamp DNA Mini Kit (Qiagen, Valencia, CA). DNA from whole blood and white blood cell buffy coats was extracted using the DNeasy Tissue Kit (Qiagen).

### *CMAH *gene analysis

The *CMAH *cDNA sequence of the domestic dog [ENSEMBL: ENSCAFT00000016708] was obtained from the ENSEMBL public database [42]. The sequence of 13 dog *CMAH *exons was queried against the cat 2× whole genome sequence database [43] in the cross-species MegaBLAST application [44]. More than one sequence was identified in the trace archive for each feline exon, thus BLAST2 [44] and MegAlign software (Madison, WI) were used to build a feline *CMAH *gene contig. PCR primers (Table 2) were developed in the intronic regions flanking each identified exon using the software Primer 3.0 [45] and Net primer [46]. Labeled, unlabelled and M13 tailed primers used in the genotyping described below, were synthesized by Operon (Huntsville, AL). Primers were tested for efficient product amplification on a DNA Engine Gradient Cycler (MJ Research, GMI, Ramsey, MN) as previously described [47]. Final reaction conditions for each primer pair were as follows: 1pmol of each forward and reverse primer, 1.25 mM dNTPs, 1.75 mM MgCl_2_, 1× Buffer and 0.375U of Taq (ABgene, Epsom, UK) polymerase in 30 μl reaction. PCR conditions included an initial denaturation at 95°C for 2 minutes followed by 35 cycles of 95°C for 1 minute, 58°C for 45 sec and 72°C for 45 sec with a final extension at 72°C for 10 minutes. Each exon was amplified from genomic DNA of four type A cats; a random bred cat, a Birman, a British Shorthair and a Devon Rex, and three type B cats; a Birman, a British Shorthair and a Devon Rex. The *CMAH *genomic sequence was also determined for one Ragdoll and one Sphynx with the AB blood type (Table 1). All PCR products were size separated on 2% agarose gels and the amplicons extracted and purified using the QIAquick^® ^Spin kit (Qiagen). Purified genomic products were directly sequenced using BigDye Terminator V 3.1 (Applied Biosystems, Foster City, CA, USA), purified through Sephadex 50 columns (Amersham Biosciences, Uppsala, Sweden) and electrophorectically separated on an ABI 3730 DNA Analyzer (Applied Biosystems). Sequences were verified and aligned using the software Sequencer version 4.5 (Gene Codes Corp., Ann Arbor, MI).

**Table 2:** Sequences and primers for *CMAH *analysis in the cat.

***CMAH *Region**	**Forward Primer 5' – 3'**	**Reverse Primer 5' – 3'**	**size bp**
5'UTR – e1	TTCCTTTGCTGCTGGAGTG	CAACACTGAGCAAGCAGAGC	653
Exon 2	CAACACTGAGCAAGCAGAGC	GGTTTCCTCTCTCTCAGGGG	589
Exon 3	CTCTTAGGACATGGACTGAACG	CTTTGGTAAGACGGGTGAGAG	458
Exon 5	GTGTCTGTCGCATCACCTCT	GGATGAGAAGAAGCGGACTC	668
Exon 6	GGCAGAGAGCAGGAAGACAC	TTTTCCCCCATTTACCAATG	571
Exon 7	TAGCCGATTTTTGTCTGTGC	TCAACACTTCCATACATCACCC	669
Exon 8	GATCCCATCATGCTTCCTCT	GAAACCAGACTTGCAAAGCA	630
Exon 9	GTGTCTTTGGCTGGTTTGGT	GACTTTTAGCCACGCCATTC	642
Exon 11	GCCACACAGCTCCCATAGAC	TGTTGTTGTGGGTTTTGTGG	624
Exon 12	CATGGTGTTTGCTGTCTCAT	CCTCTACATGCTTACTGCCC	647
Exon 13	GACATGAGGGGTCGGTACG	CTCAACTCCAGCGTGCATTT	579
Exon 14	TTCACTGTTGGATGATGTGG	GACTTTCAGGGGAGTGAGAAG	700
^1^cDNAe2-6	GACGACGGAGGTCTTGTTGT	TTACCCCACACTGAAGGAGC	608
^1^cDNAe5-11	CAAGAGGATGGTGTTTGACC	GCATCATAGAGCCTCCAGAG	817
^1^cDNAe9-14	GGAATCTCACCCATCAGACA	CTGTGGGTAGAGCAGACCTG	656
5' RACE		AAATACGTCAATCCCCCAGGCAGCTTCT	~500
3' RACE	AACTCCCTCTCACGCCACCCAACTG		440
Δ-53	TCCTCCGTCTCATACTTTGTGG	CATTTCCAGGGGAGCAGC	167
UTR SNPs	^2^GCCGGGTGTAAGGTTCTC	CACCTGGAGCCCAATAGG	247
^3^C-371T		AGCACGTGTAGGCT	
^3^G-217A		AAGTGTGCAGGTG	
Exon 2	^2^CTGCTGACCTGAAGGAAGGAAT	ACCCGTGAGAAGGCTCAGAA	187
^3^G139A		GGCGTGCAAGAACG	
Exon 3	TGTCAGATGCACAAAGCACAAC	^2^TGCTGTGATCGTTACTGGCGT	141
^3^T265A	TGTGAGCACCATGAAA		
Exon 10	CGACTAACTGTCACCTTTGTGTCTT	^2^TGAATGCTGCTGTAGGAGATGA	128
^3^G1266A	AAGCAAGGGCATCATA		
Exon 12	^2^ATTCGGAGCCGGGTTGAC	CCTGTAAGTTGGAAAGTGCCTG	135
^3^G1591A		GTATATAGGATTCCAAACCC	

1. Nested primers for the cDNA analysis. The positions of the primer in the exons are indicated in the primer name. 2. The M13 tail was attached to the indicated end of the indicated primer. 3. Primer for the pyrosequencing reaction of each amplified SNP region.

### *CMAH *mRNA analyses

Total RNA was extracted from whole blood samples from a cat of each blood type, including a type A Oriental Shorthair, a type B Persian, a type AB Siamese and an obligate carrier of type B (type Ab) that was a hybrid between a Persian and an Oriental Shorthair) (Table 1). RNA was extracted using the Versagene™ RNA Purification Kit (Gentra System, Minneapolis, MN) or the PAXgene™ Blood RNA System (Qiagen). Three pools of cDNA template were synthesized by reverse transcription of 1 μg of mRNA with random primers, a gene specific primer, *CMAH*_GSP_ 5'-GAGGTACGTGATCTGCACTT-3', and oligo dT primers using SuperScript III (Invitrogen, Carlsbad CA). PCR amplification of *CMAH *from the random primer pool of cDNA was performed using nested primers listed in Table 2. To obtain the 5' and 3' portions of the gene, RNA ligase-mediated rapid amplification of 5' and 3' cDNA ends (RLM-RACE) were performed using the GeneRacer™ Kit (Invitrogen). The RACE primers were also presented in Table 2. The 5' RACE used the cDNA pool generated by the *CMAH *specific primer listed above and the 3' RACE used the cDNA pool generated from the oligo dT primers. The PCR conditions included an initial denaturation for 2 min, 5 cycles at 95°C for 30 sec and 72°C for 1 min, 5 cycles at 95°C for 30 sec and 70°C for 1 min, 27 cycles at 95°C for 30 sec, 68°C for 30 sec and 72°C for 1 min, followed by a 10 min extension at 72°C. All PCR products were directly sequenced as previously described except that the 5'RACE-PCR products that were cloned using the TOPO TA Cloning Kit for Sequencing (Invitrogen) prior to sequencing. Seven to twenty-four 5' RACE cDNA clones from each of the four cats were selected and sequenced.

### Mutation genotyping

Six SNPs identified by genomic sequence analyses (Table 1) were screened across the entire sample set by pyrosequencing using an M13 biotinylated primer (5' AGCGCATAACAATTTCACAGG 3') (Table 2). Two SNPs identified in the 5' UTR were amplified in a single PCR multiplex reaction while four coding region SNPs were detected in separate pyrosequencing amplification reactions. Pyrosequencing reactions were performed on a PSQ 96MA (Biotage, Uppsala, Sweden). Amplification conditions for the pyrosequencing reactions were as follows: 2 min denaturation at 95°C followed by 45 cycles of a 45 sec denaturation at 95°C, annealing for 30 sec at 62°C and an extension at 72°C for 30 sec. The reactions were completed with a 10 min extension at 72°C. The 5' UTR 18 bp indel was genotyped as a size variant by PCR with a FAM fluorescently labeled forward primer (Table 2) in all 213 cats and electrophorectically separated on an ABI 3730 DNA Analyzer (Applied Biosystems).

## Authors' contributions

BB performed the research; TN provided the necessary sample set and blood typing data. RG assisted and mentored all laboratory research and NP conceived of the idea and wrote the paper. LM designed and performed pyrosequencing assays, MP provided study design input and manuscript editing, ML provided study design input and manuscript editing and LAL developed the study design, collaborations and wrote the paper. All authors read and approved the final manuscript.

## Supplementary Material

Additional File 1 **Sequence and protein translation of feline *CMAH***. The composite feline sequence of *CMAH *is presented with its protein translation listed above the cDNA sequence. Identified DNA variants are presented in bold and underlined. Mutations that cause an amino acid change are presented with an underlined codon, the mutation and protein code in bold. The cDNA sequence of the dog [Ensembl: ENSCAFT00000016708], human [GenBank: AF074480] and mouse [GenBank: D21826] are presented below the cat. Missing sequences are presented as dashes. The 2 codons of exon 1 have not yet been identified in the dog, 2 additional codons are absent downstream. Approximately 31 amino acids are deleted in humans, causing inactivation of the enzyme. Click here for file
