# Supplementary material for: Cytidine monophospho-N-acetylneuraminic acid hydroxylase (CMAH) mutations associated with the domestic cat AB blood group
Source: BMC Genet. 2007 Jun 6;8:27. doi: 10.1186/1471-2156-8-27 (PMC1913925; doi:10.1186/1471-2156-8-27)
Supplement: Additional File 1 — Sequence and protein translation of feline CMAH. The composite feline sequence of CMAH is presented with its protein translation listed above the cDNA sequence. Identified DNA variants are presented in bold and underlined. Mutations that cause an amino acid change are presented with an underlined codon, the mutation and protein code in bold. The cDNA sequence of the dog [Ensembl: ENSCAFT00000016708], human [GenBank: AF074480] and mouse [GenBank: D21826] are presented below the cat. Missing sequences are presented as dashes. The 2 codons of exon 1 have not yet been identified in the dog, 2 additional codons are absent downstream. Approximately 31 amino acids are deleted in humans, causing inactivation of the enzyme. [file 1471-2156-8-27-S1.pdf]

Additional file 1: Sequence and protein translation of feline *CMAH*

The composite feline sequence of *CMAH* is presented with its protein translation listed above the cDNA sequence. Identified DNA variants are presented in bold and underlined. Mutations that cause an amino acid change are presented with an underlined codon, the mutation and protein code in bold. The cDNA sequence of the dog [Ensembl: ENSCAFT00000016708], human [GenBank: AF074480] and mouse [GenBank: D21826] are presented below the cat. Missing sequences are presented as dashes. The 2 codons of exon 1 have not yet been identified in the dog, 2 additional codons are absent downstream. Approximately 31 amino acids are deleted in humans, causing inactivation of the enzyme.

(Please see the next page).

```
            1.......10........20........30........40........50........60........70.....
Protein       M  G  S  I  E  Q  T  T  E  V  L  L  C  L  S  P  G  E  A  A  D  L  K  E  G
Cat         ATGGGCAGCATTGAACAGACGACGGAGGTCTTGTTGTGTYTGTCACCTGGAGAAGCTGCTGACCTGAAGGAAGGA
Dog         ------...........A..A..A...A..C.C...CA.........CATT....T...CA.T............
Human       ...........C.....A..A..T...A..C.......C..A.......TT....T...CAGT..T.........
Mouse       ...ATGGA..GGA.......AG.T...A..C..C..ACC.....T....CT....T...CA....C........G
            ...80........90.......100.......110.......120.......130.......140.......150
Protein       I  N  F  L  R  N  K  K  T  G  K  D  F  I  L  Y  K  S  K  N  R  V  R  A  C
Cat         ATCAATTTCTTGAGAAACAAGAAGRCCGGCAAAGATTTCATTCTGTACAAGAGCAAGAATCGCRTGAGGGCGTGC
Dog         ...........TC....T.....C..T..........A...C..C.......AT.......A.C..C....A...
Human       ...........TC.C..T....GC..T........C.A.G.CT.........AT....GC..AC.......A...
Mouse       ........T..TC....T....CT..T..G.....G.A....T.A......GAG...G.C.ATC.A.A...A...

            .......160.......170.......180.......190.......200.......210.......220.....
Protein       K  N  V  C  K  H  Q  G  G  L  F  I  K  D  I  E  D  L  D  G  R  S  V  R  C
Cat         AAGAACGTGTGCAAGCATCAAGGAGGCCTGTTCATAAAAGACATCGAAGATTTAGACGGAAGGTCTGTCAGATGC
Dog         .....TA........................................G........T...........T......
Human       .....TA..........C.......................T.....G.......C......-------------
Mouse       ......C.C........C..G..............G...........G........T........C..T.A....

            ..230.......240.......250.......260.......270.......280.......290.......300
Protein       T  K  H  N  W  R  L  D  V  S  T  M  K  Y  V  N  P  P  G  S  F  C  Q  D  E
Cat         ACAAAGCACAACTGGAGGTTGGATGTGAGCACCATGAAAWACGTCAATCCCCCAGGCAGCTTCTGTCAAGACGAA
Dog         .....A..........A...A.................G..TA....C......A................T...
Human       ---------------------------------------------------------------------------
Mouse       ................A...A..C.................TA....C..T.....G.................G

            .......310.......320.......330.......340.......350.......360.......370.....
Protein       L  V  V  E  M  D  E  E  N  G  L  L  L  L  E  L  N  P  P  N  P  W  D  S  E
Cat         CTAGTTGTTGAAATGGATGAAGAMAATGGACTTTTACTTCTAGAACTGAATCCTCCCAATCCCTGGGATTCAGAA
Dog         ........................................................T..C...............
Human       ----.................A.C..C........G...T................T..C..T.....C.T.C.G
Mouse       ..C...A..............A.C.....G....CC..GG..........C.....T..C........C..T..T
```

```
                ..380.......390.......400.......410.......420.......430.......440.......450
Protein          P  R  S  P  E  H  L  A  F  G  E  V  Q  I  T  Y  L  T  H  A  C  M  D  L  K
Cat             CCCAGATCTCCTGAACATTTGGCTTTTGGAGAAGTGCAGATCACGTACCTCACCCACGCCTGCATGGACCTCAAG
Dog             ......G...G......................A.A------.........G.T........T............
Human           ...............G.G.................A.....A..A..T.....T..T..................
Mouse           .....G.........G.A..A........G.....A.....A..A..T.....T..T..................

                .......460.......470.......480.......490.......500.......510.......520.....
Protein          L  G  D  K  R  M  V  F  D  P  W  L  T  G  P  A  F  A  R  G  W  W  L  L  H
Cat             CTGGGAGACAAGAGGATGGTGTTTGACCCCTGGTTAACGGGTCCTGCGTTCGCCCGAGGCTGGTGGCTGCTGCAC
Dog             .......................C.............TA........C..T.....G..A...............
Human           T.A...........A..............T.......TC........T..T.....T..A......T....C..T
Mouse           T...........C.A.....A........T.......TT..C.....T..T........A......T....A..T

                ..530.......540.......550.......560.......570.......580.......590.......600
Protein          E  P  P  S  D  W  L  E  R  L  C  Q  A  D  L  I  Y  I  S  H  M  H  S  D  H
Cat             GAGCCTCCGTCTGATTGGCTGGAGAGGCTGTGCCAGGCAGACCTGATTTACATCAGTCACATGCACTCAGACCAC
Dog             ........C................A..................A..............T........G.....T
Human           ........A...................................C..............TC..............
Mouse           ........A.....C...T..............A.A........C.....T.....C..................

                .......610.......620.......630.......640.......650.......660.......670.....
Protein          L  S  Y  P  T  L  K  E  L  A  G  R  R  P  D  I  P  I  Y  V  G  N  T  E  R
Cat             CTGAGTTACCCCACACTGAAGGAGCTGGCTGGGAGAAGACCGGATATTCCCATTTATGTTGGAAACACGGAAAGA
Dog             ..C..C.....G.........A....T....A.........A.................................
Human           ....................AA....T..............A..........................A.....G
Mouse           .....C.....T..C......C....TT.CCA....C....A..C.................CG....A.....G

                ..680.......690.......700.......710.......720.......730.......740.......750
Protein          P  V  F  W  N  L  N  Q  S  G  V  Q  L  T  N  I  N  I  V  P  F  G  I  W  Q
Cat             CCTGTATTTTGGAATCTGAATCAGAGCGGTGTCCAGTTGACCAATATCAATATAGTGCCATTCGGAATATGGCAG
Dog             .........................................T.........G..........T............
Human           .........................................T.........G.C........T............
Mouse           .....G........C...G.......T..C...GG...A..T..C.....CG.G..T.....T...........A
```

```
            .......760......770......780......790......800......810......820.....
Protein       Q  V  D  K  N  L  R  F  M  I  L  M  D  G  V  H  P  E  M  D  T  C  I  I  V
Cat         CAGGTAGACAAAAATCTTCGATTCATGATCTTGATGGATGGCGTTCATCCTGAGATGGACACTTGCATTATTGTG
Dog         ..........................................A................................
Human       .....G................................C..T.................................
Mouse       .............G...G..G.................C.......................A........C...

            ..830......840......850......860......870......880......890......900
Protein       E  Y  K  G  H  K  I  L  N  T  V  D  C  T  R  P  N  G  G  R  L  P  E  K  V
Cat         GAGTATAAAGGTCATAAGATACTTAATACAGTGGACTGCACCAGACCCAATGGGGGAAGGTTGCCTGAGAAGGTT
Dog         ...........................................................................
Human       .....C...........A.....C....T...A...........................C.....AT.......
Mouse       .....C...........A.....C..C.................................C.T........A...

            .......910......920......930......940......950......960......970.....
Protein       A  L  M  M  S  D  F  A  G  G  A  S  G  F  P  M  T  F  S  G  G  K  F  T  E
Cat         GCTCTAATGATGAGTGATTTTGCTGGAGGAGCATCAGGCTTTCCAATGACTTTCAGTGGTGGAAAATTTACCGAG
Dog         .......................................................................T...
Human       .......................................................................G...
Mouse       ....................C..A.....T.........................................T...

            ..980......990.....1000.....1010.....1020.....1030.....1040.....1050
Protein       E  W  K  A  Q  F  I  K  A  E  R  K  K  L  L  N  Y  K  A  R  L  V  K  D  L
Cat         GAATGGAAAGCCCARTTCATTAAAGCAGAGAGGAAGAAGCTCTTGAACTACAAGGCCCGGCTGGTGAAGGACCTA
Dog         ............G.G........GA....A.......................A.....................
Human       ........................A....A............C.............T............A....G
Mouse       ..............G........G..T..A..A.GA.....TC....T.....A..T.A...............G

            ......1060.....1070.....1080.....1090.....1100.....1110.....1120.....
Protein       Q  P  R  I  Y  C  P  F  A  G  Y  F  V  E  S  H  P  S  D  K  Y  I  K  E  T
Cat         CAACCCCGAATTTATTGCCCCTTTGCTGGGTATTTTGTGGAATCTCACCCATCAGACAAGTATATTAAGGAAACA
Dog         .....T.....C.....T..........................C..T...........................
Human       .................T............................................C..C.........
Mouse       ..G........C..C..T..G...........C........G...........T........C............
```

```
           .1130......1140......1150......1160......1170......1180......1190......1200
Protein      N  I  K  N  D  P  N  E  L  N  N  L  I  K  K  N  S  D  V  V  T  W  T  P  R
Cat        AACATCAAAAATGACCCAAATGAACTCAATAATCTTATCAAGAAAAACTCTGATGTGGTAACGTGGACCCCACGG
Dog        .......................G..T..............................A....A...........A
Human      ....C........................C...........................A....A........T..A
Mouse      ....C................C.G.....C..........G............C.....G..A...........A

           ......1210......1220......1230......1240......1250......1260......1270.....
Protein      P  G  A  T  L  D  L  S  R  M  L  K  D  P  T  D  S  K  G  I  I  E  P  P  E
Cat        CCTGGAGCCACTCTTGATCTGAGTCGGATGCTAAAGGACCCGACAGACAGCAAGGGCATCATAGARCCTCCAGAG
Dog        ........................A................A..T....................G......A.T
Human      ..G........C.........G.AA.A.....G.....T.GA.......................G.........
Mouse      .....C..TGTC..C..C..TG.CA.......G........A.................TG.G..G.........

           .1280......1290......1300......1310......1320......1330......1340......1350
Protein      G  T  K  I  Y  K  D  S  W  D  F  E  P  Y  L  N  I  L  N  A  A  V  G  D  E
Cat        GGGACCAAGATCTACAAGGATTCCTGGGACTTTGAACCCTATTTGAACATCTTGAATGCTGCTGTAGGAGATGAG
Dog        .....A..A..T......................G...A........AG.............A....A......A
Human      .....A..A..T.................T........T......GAA...............C..........A
Mouse      .....A.....T......................GC..G..CC..G.G.........T.......CA.......A

           ......1360......1370......1380......1390......1400......1410......1420.....
Protein      I  F  L  H  S  S  W  I  K  E  Y  F  T  W  A  G  F  K  D  Y  N  L  V  V  R
Cat        ATATTTCTTCACTCATCCTGGATAAAAGAATACTTCACYTGGGCKGGATTTAAGGATTACAACCTGGTGGTCAGG
Dog        ................................T.....T.....A..............T...............
Human      ......................................T.....T........A...........T.........
Mouse      ..C..CTG...T...........T.....G........G.....T.........A....................

           .1430......1440......1450......1460......1470......1480......1490......1500
Protein      M  I  E  T  D  E  D  F  S  P  F  P  G  G  Y  D  Y  L  V  D  F  L  D  L  S
Cat        ATGATTGAAACAGATGAGGACTTCAGCCCTTTTCCYGGAGGATATGACTATTTGGTTGACTTTCTAGACTTATCC
Dog        ............A..................................................T....T......
Human      ........G................AT....................................T....T......
Mouse      .................A..T....................G..C......C....G...........T......
```

```
               ......1510......1520......1530......1540......1550......1560......1570.....
Protein         F  P  K  E  R  P  S  R  E  H  P  Y  E  E  I  R  S  R  V  D  V  I  R  H  V
Cat            TTTCCAAAAGAGAGACCAAGCCGGGAACACCCGTATGAGGAAATTCGGAGCCGGGTTGACGTCATCAGACACGTG
Dog            ...........A..............G...T............................T.........T.T...
Human          ..C........A......CAA..A.....T..C...........C.AT........G..T...............
Mouse          .....G.....A.....C........G..T..T.....A.....C.AT........G..T........GT.....

               .1580......1590......1600......1610......1620......1630......1640......1650
Protein         V  K  N  G  L  L  W  D  D  L  Y  I  G  F  Q  T  R  L  Q  R  S  P  D  I  Y
Cat            GTAAAGAACGGTCTTCTCTGGACRACTTGTATATAGGATTCCAAACCCGGCTGCAACGGAGTCCTGACATATAC
Dog            ..G..A..T.....A........T................................G...GA.............
Human          ..G.....T.....A........T..G....................A.....C..G...GA.............
Mouse          ..G........C..G..G.....T..TC.......T........G.....AT...TG...GAC.....T......

               ......1660......1670......1680......1690......1700......1710......1720.....
Protein         H  H  L  F  W  N  H  F  Q  I  K  L  P  L  T  P  P  N  W  R  S  F  L  M  H
Cat            CATCACCTGTTTTGGAACCATTTTCAAATAAAACTCCCTCTCACGCCACCCAACTGGAGGTCCTTCCTGATGCAC
Dog            .....T...................................A.T......................T........
Human          .................T....................C.....A.............A...A.........TG.
Mouse          .....T...........T........G..............A..A.............A...G.....A......

               ..380.......390.......400.......410.......420.......430.......440.......450
Protein         C  G  *
Cat            TGTGGGTAG
Dog            .........
Human          ....A.C..AATGGGCCTGCGATTTTGCAATTCTCTACAGAAAGAA
Mouse          ....AT...
```
